# Study on the Effects of Tannase on the De Astringency of Pomegranate Juice

**DOI:** 10.3390/foods14060985

**Published:** 2025-03-14

**Authors:** Guida Zhu, Longwen Wang, Han Wang, Zihan Chen, Xue Li, Yi Ji, Jing Yu, Ping Song

**Affiliations:** Department of Food Science and Engineering, Nanjing Normal University, No.1 Wenyuan Road, Nanjing 210023, China; 232702011@njnu.edu.cn (G.Z.); 232712052@njnu.edu.cn (L.W.); 232712036@njnu.edu.cn (H.W.); 232712026@njnu.edu.cn (Z.C.); 232712032@njnu.edu.cn (X.L.); 242712073@njnu.edu.cn (Y.J.)

**Keywords:** tannase, punicalagin, astringency, antioxidant, polyphenol

## Abstract

Reducing the punicalagin content is an effective strategy for eliminating the astringency of pomegranate juice. In this study, pomegranate juice was used as the raw material, and tannase was applied to convert punicalagin into ellagic acid and gallic acid. The effects of tannase concentration, reaction time, and temperature on juice deastringency were evaluated, along with the antioxidant and physicochemical properties of the treated juice. The results demonstrated that, under optimal conditions (33.9 U/100 mL tannase, 30 °C, 90 min reaction time), the punicalagin content decreased by 27.8%, while the ellagic acid and gallic acid levels increased by 24.2% and 32.3%, respectively, effectively reducing the juice’s astringency. Under these conditions, the total phenolic content reached 110 mg/100 g, with a free radical scavenging capacity of 69.8%, significantly enhancing the juice’s antioxidant properties. These results suggest that tannase treatment of pomegranate juice enhances the polyphenol content, thereby improving its health benefits without compromising the product quality.

## 1. Introduction

The pomegranate is extensively cultivated worldwide, with a rich diversity of germplasm resources. Pomegranates are typically consumed fresh or commercially processed into juice, making them one of the most widely consumed fruits globally. Pomegranate juice exhibits various health benefits, including antioxidant, anticancer, anti-inflammatory, vision-enhancing, and cosmetic properties [1,2,3,4]. As a result, pomegranate juice has increasingly gained popularity as a premium beverage in both domestic and international markets [5].

Pomegranate peels and seeds are rich in tannins, which can be introduced into the juice during extraction due to the incomplete removal of these parts. This incorporation of tannins leads to a bitter and astringent taste, negatively impacting the quality of the final product [6]. Punicalagin, the main ellagitannin in pomegranate, is a type of ellagitannin widely found in plants and is the primary source of the astringency in pomegranate juice. Therefore, the removal of punicalagin from the juice is of significant importance in improving the quality of pomegranate juice. Traditionally, the removal of astringency from fruits was achieved by treating them with alcohol or dry ice. However, given the large quantities of pomegranates harvested within a short period, these time-consuming and labor-intensive methods hinder the production of pomegranate juice. In recent years, researchers have focused on developing methods to remove tannins from fruit juices, effectively eliminating astringency while preserving the original flavor of the juice. Liu et al. [7] employed a chemical precipitation method using ginger protein as a tannin remover, effectively eliminating tannins from rose hip juice. Zhang et al. [8] used gelatin to remove tannins from pomegranate juice. However, both chemical precipitation and gelatin methods are susceptible to the loss of nutrients and flavor compounds, whereas tannase enzyme treatment is highly selective and does not involve harmful substances. But there are no reports on the use of tannase for the removal of punicalagin from pomegranate juice.

Tannase, also known as tannin acyl hydrolase (TAH), can hydrolyze the ester bonds in ellagitannins [9], thereby breaking down punicalagin and releasing low-molecular-weight polyphenolic compounds such as ellagic acid and gallic acid [10]. Phenolic acids are essential bioactive compounds in fruit juices, contributing significantly to their antioxidant properties, flavor profile, and health benefits [11]. These compounds, including gallic acid, ellagic acid, caffeic acid, and ferulic acid [12], play a crucial role in neutralizing free radicals, preventing oxidative stress, and enhancing the stability of the juice [13]. Their strong antioxidant activity not only provides health benefits [14] but also helps protect the juice from oxidative deterioration, preserving its sensory and nutritional qualities over time [15]. To accurately quantify and characterize these phenolic compounds, various analytical techniques are employed [16]. High-Performance Liquid Chromatography (HPLC) is the most widely used method due to its high sensitivity, precision, and ability to separate complex mixtures [17]. In particular, HPLC coupled with diode array detection (HPLC-DAD) or mass spectrometry (HPLC MS) [18] allows for the precise identification and quantification of phenolic acids in fruit juices. Other advanced techniques [19,20], such as ultra-high-performance liquid chromatography (UHPLC), gas chromatography-mass spectrometry (GC-MS), and capillary electrophoresis (CE) [21,22], can also be used depending on the chemical nature of the compounds and the required sensitivity. Ensuring a high concentration of phenolic acids in juice is essential for maintaining its antioxidant capacity and prolonging its shelf life. By preventing oxidative degradation, these bioactive molecules help retain the juice’s natural color, taste, and nutritional value, making them vital components for both consumer health and product stability.

Therefore, the use of tannase to hydrolyze punicalagin in pomegranate juice, releasing ellagic acid and gallic acid to reduce juice astringency, holds promising prospects. Tannase is widely distributed among various animals, plants, and microorganisms, particularly in fungi of the genera Aspergillus and Penicillium. Furthermore, tannase production has been reported in Trichoderma, yeast, as well as certain bacteria and actinomycetes [23,24]. Presently, most tannase available on the market is of a microbial origin, as microbial tannase typically demonstrates superior activity and selectivity, and can be produced consistently on a large scale [25,26].

In our previous work, the target strain was selected from soil, and fermentation conditions were optimized using response surface methodology to enhance the tannase production capacity of the strain. Through the adaptation of the tannase-producing strain, the enzyme activity reached approximately 15 U/mL [27]. In this study, we employed a tannase-producing strain and optimized the enzymatic hydrolysis conditions for punicalagin, focusing on the key factors such as hydrolysis time, temperature, and enzyme activity. Based on the optimized conditions, we measured the ascorbic acid (VC) content, total phenolic content (TPC), and DPPH radical scavenging capacity of pomegranate juice at different enzymatic hydrolysis times. By applying tannase to treat raw pomegranate juice from Xinjiang, we aimed to identify the optimal conditions for astringency removal, providing a technical reference and support for improving the quality of pomegranate juice products.

## 2. Materials and Methods

### 2.1. Chemicals

Tannic acid, gallic acid, propyl gallate, methanol, rhodanine, citric acid, sodium citrate, potassium hydroxide, Fast Blue Salt B, Folin-Ciocalteu reagent, acetic acid, EDTA, DPPH, and Na_2_CO_3_ were all purchased from Shanghai Macklin Biochemical Technology. Czapek-Dox agar and Czapek-Dox broth medium were purchased from Qingdao Hope Bio-Technology (Qingdao, China).

A tannase-producing strain was isolated from the soil beneath pomegranate trees in Nanjing, Jiangsu Province. Based on its morphological characteristics and ITS molecular sequence analysis, the strain was identified as Aspergillus flavipes and is currently preserved at the Guangdong Microbial Culture Collection Center.

### 2.2. Production and Activity Assay of Tannase

**Figure 1:**
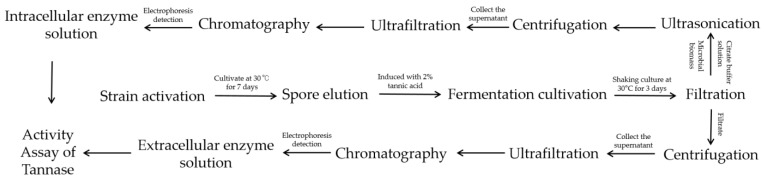
The production process of tannase and the determination of its enzymatic activity.

The cryopreserved strain was first activated on Czapek medium. Spores were washed off with sterile water, and the resulting spore suspension was inoculated into the seed medium at a 1% inoculation volume. Intracellular and extracellular enzyme solutions were then prepared through filtration, centrifugation, and ultrasonic disruption. The detailed production process of tannase is illustrated in Figure 1. Tannase activity was assessed using the methanol-rhodanine method [28,29], yielding a regression equation of Y = 0.0006X − 0.0328 (R^2^ = 0.9939). The activity of extracellular tannase was calculated as 15.4 U/mL, while intracellular tannase activity was 1.2 U/mL. Given the substantial difference in activity levels, only the extracellular enzyme was selected for further experiments, following verification using single-factor tests.

The enzymatic activity of tannase exhibits distinct stability profiles under different temperature conditions. At 30 °C and pH 5, tannase exhibited high enzymatic activity within the initial 8 h and maintained relatively high activity for up to 48 h. When stored at 4 °C, the enzyme retained its activity for up to one month. Freezing at −20 °C significantly enhanced its stability, allowing it to maintain high activity for over six months. Under these frozen conditions, tannase has been shown to preserve its enzymatic activity for extended periods of up to 12 months or longer, demonstrating exceptional long-term stability.

### 2.3. Preparation Process of Pomegranate Juice

The pomegranate fruits used in this study were purchased fresh from a local market. A single 25 kg batch of plump, undamaged fruits was obtained to provide uniform raw material for all the experiments. The preparation of pomegranate juice involved several key steps. First, a portion of pomegranate fruits was selected, washed thoroughly, and then juiced while retaining 30% of the aril membrane and peel by weight. The juice was filtered to obtain fresh pomegranate juice, which was then transferred into bottles, sealed, labeled, and stored at 4 °C for later use. For all the enzymatic hydrolysis experiments, 100 mL of pomegranate juice was used, and tannase was added before incubation in a shaking incubator at various temperatures. After the specified hydrolysis time, the enzyme was inactivated by heating to 90 °C for 5 min to stop the reaction. After cooling to room temperature, 10 mL of the pomegranate juice was transferred to a 15 mL centrifuge tube for subsequent analysis. Sensory evaluation of the pomegranate juice was performed for each sample, followed by further analysis or processing. A single-factor experimental design was used to systematically analyze the effects of the hydrolysis conditions on the punicalagin, ellagic acid, and gallic acid content in pomegranate juice. The enzyme concentration, hydrolysis temperature, and time were treated as independent variables, while other parameters, such as enzyme type, were held constant. All the experiments were conducted in triplicate.

### 2.4. UPLC Detection of Punicalagin, Ellagic Acid, and Gallic Acid

Add 100 mL of pomegranate juice to an Erlenmeyer flask, followed by the addition of tannase. Incubate the mixture in a shaker. At specified time intervals, withdraw 10 mL of pomegranate juice into a 15 mL centrifuge tube and stand by. Perform sensory evaluations of the pomegranate juice at each time point. From the 15 mL centrifuge tube, transfer 2 mL of fresh pomegranate juice into a 2 mL centrifuge tube and centrifuge at 10,000 rpm for 1 min. Collect 1 mL of the supernatant and add 400 µL each of 300 g/L zinc sulfate solution and 106 g/L potassium ferrocyanide solution, mixing thoroughly via vortexing. Centrifuge again at 10,000 rpm for 1 min. Filter the supernatant through a 0.22 µm membrane filter and measure the concentrations of punicalagin, ellagic acid, and gallic acid.

The analysis was conducted using a Shimadzu LC-40 Ultra-High Performance Liquid Chromatograph (Shimadzu Corporation, Shanghai, China) equipped with a Sepax Bio-C18 column (Sepax Technologies, Shanghai, China) with dimensions of 4.6 mm in diameter and 250 mm in length. The column temperature was maintained at 30 °C. The mobile phase consisted of A: 0.2% phosphoric acid and B: pure methanol [30]. The flow rate was set at 0.6 mL/min, with an injection volume of 2 µL. The gradient elution program was as follows: 0 min, 2% B; 10 min, 5% B; 15 min, 50% B; 25 min, 80% B; 30 min, 90% B; 32 min, 100% B; 35 min, 2% B. The detection wavelength for punicalagin was set at 371 nm, while ellagic acid and gallic acid were detected at 254 nm [31,32].

### 2.5. Optimization of the Pectinase Clarification Process for Pomegranate Juice Using Orthogonal Experimentation

Based on the results of single-factor experiments, the enzyme dosage, enzymatic hydrolysis time, and enzymatic hydrolysis temperature were selected as the main influencing factors. The content of punicalagin was used as the primary evaluation index, along with the concentrations of ellagic acid and gallic acid in the pomegranate juice. An L9 (3^4^) orthogonal experiment was conducted to identify the optimal clarification process. The factors and levels for the orthogonal experiment are shown in Table 1. After the reaction completed, the pomegranate juice was immersed in boiling water for 10 min to pasteurize and inactivate residual enzymes and cooled in an ice water bath.

### 2.6. Rational Antioxidant Activity Index

The content of ascorbic acid (VC) in juice was determined using the spectrophotometric method [33,34]. A 10–20 mL sample of pomegranate juice was transferred into a 100 mL amber volumetric flask, followed by the addition of 5 mL of 2 mol/L acetic acid. The solution was then diluted to the mark with distilled water and filtered. A 10 mL aliquot of the filtrate was mixed with 0.5 mL of 0.1 mol/L (EDTA) solution, 0.5 mL of 0.5 mol/L acetic acid, and 1.2 mL of 0.2% Fast Blue Salt B. The mixture was allowed to stand for 20 min before its absorbance was measured at 295 nm using a spectrophotometer. The ascorbic acid content was expressed in g/100 mL.

The total phenolic content (TPC) was measured using the Folin–Ciocalteu method [35]. Under alkaline conditions, phenolic compounds are oxidized to phenolic salt, forming a blue complex. A 1.5 mL aliquot of Folin–Ciocalteu reagent was added to 300 μL of the sample, followed by 1.2 mL of sodium carbonate solution. The mixture was then incubated in the dark for 90 min. The absorbance was measured at 765 nm using a UV spectrophotometer at 15 min intervals, with each sample analyzed in triplicate. The TPC was quantified based on the standard absorption curve and expressed as gallic acid equivalents (mg GAE/100 g).

The antioxidant activity was assessed using the DPPH assay [36]. A 0.2 mL aliquot of the sample was mixed with 1.8 mL of 0.004% DPPH-methanol solution and incubated at room temperature (25 ± 1 °C) for 30 min. The absorbance was then measured at 517 nm and recorded as AA. A blank control, consisting of 0.004% DPPH-methanol solution without the sample, was measured under the same conditions, with its absorbance recorded as AC. Each experiment was performed in triplicate. The DPPH radical scavenging activity was calculated using Equation (1).

DPPH Radical Scavenging Activity/% = (1 − AA/AC) × 100(1)

where AA is the absorbance of the sample solution and AC is the absorbance of the blank.

### 2.7. Sensory Evaluation

A panel of 10 evaluators, comprising faculty members and students specializing in food science, conducted the assessment using a descriptive sensory analysis method. The pomegranate juice was evaluated based on four attributes: color, taste, flavor, and overall acceptability, with a maximum score of 10 points for each attribute. The sensory evaluation criteria are detailed in Table 2.

## 3. Results and Discussion

### 3.1. Single-Factor Experiment

**Figure 2:**
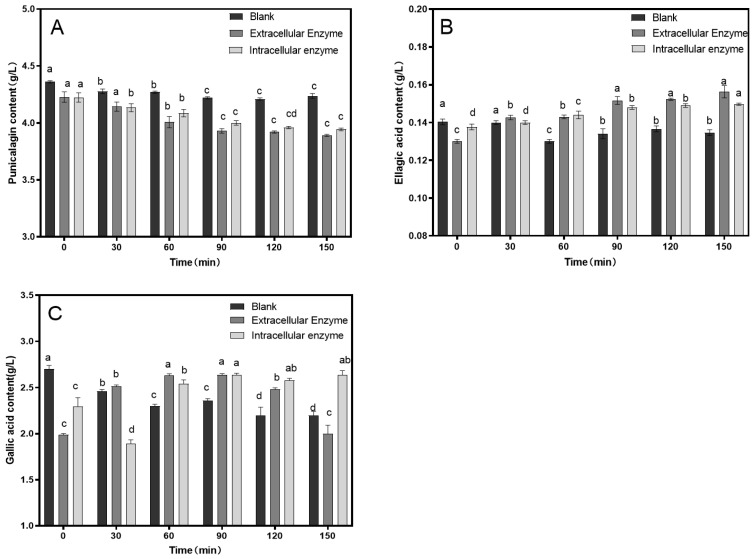
Effect of enzymatic hydrolysis time on the contents of punicalagin (**A**), ellagic acid (**B**), and gallic acid (**C**). Different lowercase letters above the bars (a, b, c, d) indicate statistically significant differences between samples. The significance level was set at 0.05.

Based on the preliminary experiments, enzymatic hydrolysis was conducted at 30 °C with an enzyme dosage of 15.4 U/100 mL to assess the impact of hydrolysis time on the astringency removal in pomegranate juice. As shown in Figure 2, the punicalagin content in the control group remained relatively unchanged over time. In the enzyme-treated group, the punicalagin content decreased significantly before 90 min of hydrolysis but showed no further significant reduction after 90 min, with the lowest punicalagin content reaching 3.89 g/L. In the control group, the ellagic acid content slightly decreased, whereas in the enzyme-treated group, the ellagic acid content increased significantly, peaking at 0.16 mg/L. The trend for gallic acid content mirrored that of ellagic acid, increasing from 1.8 g/L to 2.6 g/L.

**Figure 3:**
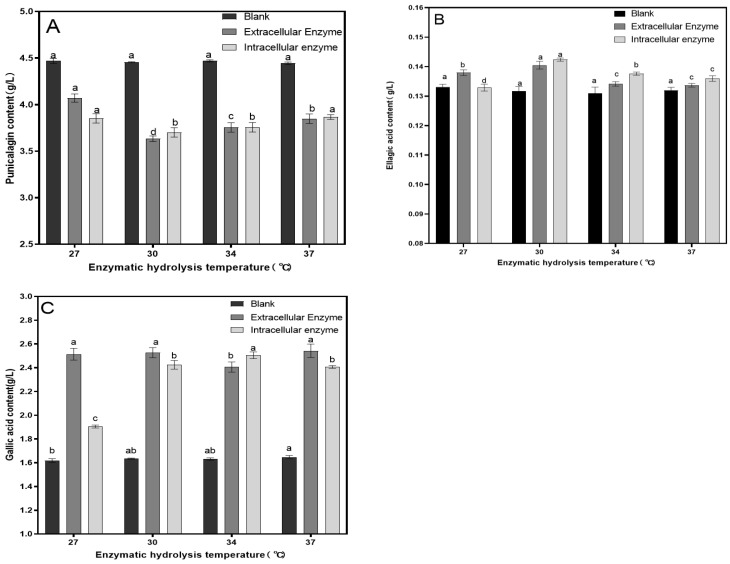
Effect of enzymatic hydrolysis temperature on the contents of punicalagin (**A**), ellagic acid (**B**), and gallic acid (**C**). Different lowercase letters above the bars (a, b, c, d) indicate statistically significant differences between samples. The significance level was set at 0.05.

A fixed amount of tannase (15.4 U/100 mL) was used for enzymatic hydrolysis for 90 min to investigate the effect of different temperatures on the removal of astringency in pomegranate juice. As shown in Figure 3, the reduction in punicalagin content was most pronounced at 30 °C, with the lowest punicalagin content decreasing to 3.65 mg/L. As the temperature increased further, the reduction in punicalagin content slowed. This is because the effectiveness of tannase increases with temperature, but excessive temperatures can negatively impact the enzyme activity. At 30 °C, the ellagic acid content increased to a maximum of 0.142 mg/L, and the gallic acid content increased to a maximum of 2.55 g/L. Considering the contents of punicalagin, ellagic acid, and gallic acid in the pomegranate juice, 30 °C was selected as the optimal temperature for enzymatic hydrolysis.

**Figure 4:**
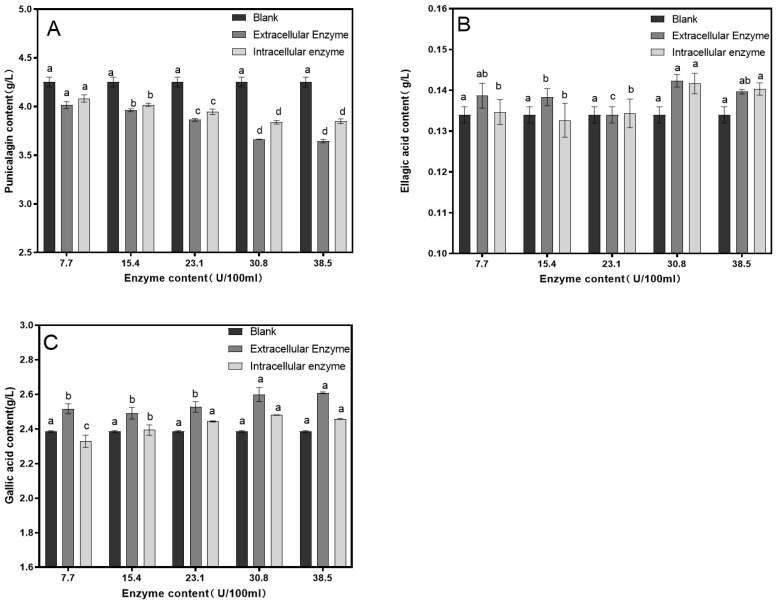
Effect of tannase concentration on the contents of punicalagin (**A**), ellagic acid (**B**), and gallic acid (**C**). Different lowercase letters above the bars (a, b, c, d) indicate statistically significant differences between samples. The significance level was set at 0.05.

Different concentrations of tannase were added to pomegranate juice and subjected to enzymatic hydrolysis at 30 °C for 90 min to assess the impact of tannase concentration on astringency removal. As shown in Figure 4, the reduction in the punicalagin content was most pronounced when the tannase concentration was 30.8 U/100 mL, with the lowest punicalagin content decreasing to 3.559 mg/L. Increasing the enzyme concentration further did not result in a significant additional decrease in the punicalagin content. The ellagic acid content increased to a maximum of 0.14 mg/L, while the gallic acid content increased to 2.6 g/L. It was observed that the extracellular enzyme had a similar effect to that of the intracellular enzyme under the same conditions; therefore, only the extracellular enzyme was selected for the subsequent orthogonal experiments.

### 3.2. Orthogonal Experimental

The range value (R) was determined by calculating the mean value (k) for each factor. A comparison of the R values was then conducted to analyze the significance of different influencing conditions. As shown in Table 3, based on the comparison of R values, the factors influencing the punicalagin content in pomegranate juice are ranked in the order of A > B > C, indicating that the enzymatic hydrolysis time has the most significant impact, followed by hydrolysis temperature, with tannase concentration having the least effect. Considering the punicalagin content as the primary evaluation index, the optimal conditions for tannase-mediated punicalagin degradation were determined to be A3B2C3. For the ellagic acid content, the order of influence is C > A > B, meaning that the tannase concentration has the greatest effect, followed by the hydrolysis time, with the hydrolysis temperature having the smallest impact. Using the ellagic acid content as the evaluation index, the optimal conditions for tannase-mediated ellagic acid production were A2B2C3. Regarding the gallic acid content, the order of influence is A > C > B, showing that the enzymatic hydrolysis time has the greatest effect, followed by the tannase concentration, with the hydrolysis temperature having the least influence. Considering the gallic acid content as the evaluation index, the optimal conditions for tannase-mediated gallic acid production were A3B1C3.

Verification experiments were conducted three times under the A3B2C3 conditions, resulting in a punicalagin content of 3.646 mg/L, ellagic acid content of 0.140 mg/L, and gallic acid content of 2.446 mg/L in the pomegranate juice. Another set of three verification experiments under A2B2C3 conditions resulted in a punicalagin content of 3.345 mg/L, ellagic acid content of 0.141 mg/L, and gallic acid content of 2.576 mg/L. Another set of three verification experiments under A3B1C3 conditions resulted in a punicalagin content of 3.595 mg/L, ellagic acid content of 0.138 mg/L, and gallic acid content of 2.546 mg/L. Compared to the eight commercial fruit juices, both ellagic acid and gallic acid were present at relatively high levels [37]. Ultimately, the optimal tannase treatment conditions for astringency removal in pomegranate juice were determined to be A2B2C3, which corresponds to a hydrolysis time of 90 min, a hydrolysis temperature of 30 °C, and a tannase concentration of 33.9 U/100 mL.

**Figure 5:**
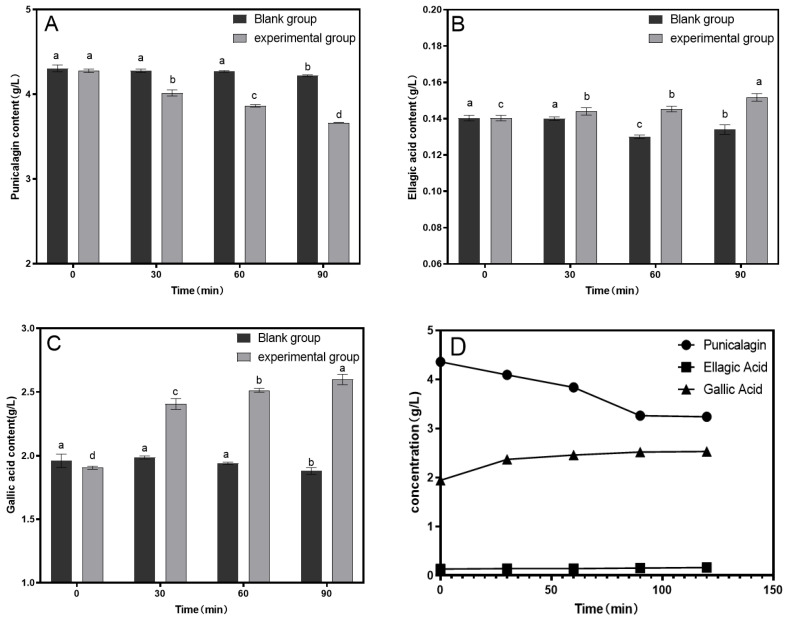
A comparative analysis of the contents of punicalagin (**A**), ellagic acid (**B**), and gallic acid (**C**) between the blank and experimental groups under optimal experimental conditions. (**D**) Changes in punicalagin, ellagic acid, and gallic acid content under optimal conditions. Different lowercase letters above the bars (a, b, c, d) indicate statistically significant differences between samples. The significance level was set at 0.05.

As shown in Figure 5, under optimal conditions, the punicalagin content in the experimental group decreased by 27.8%, whereas the ellagic acid and corilagin contents increased by 24.2% and 32.3%, respectively, compared to the blank group.

### 3.3. The Results of the Antioxidant Capacity Measurements

**Figure 6:**
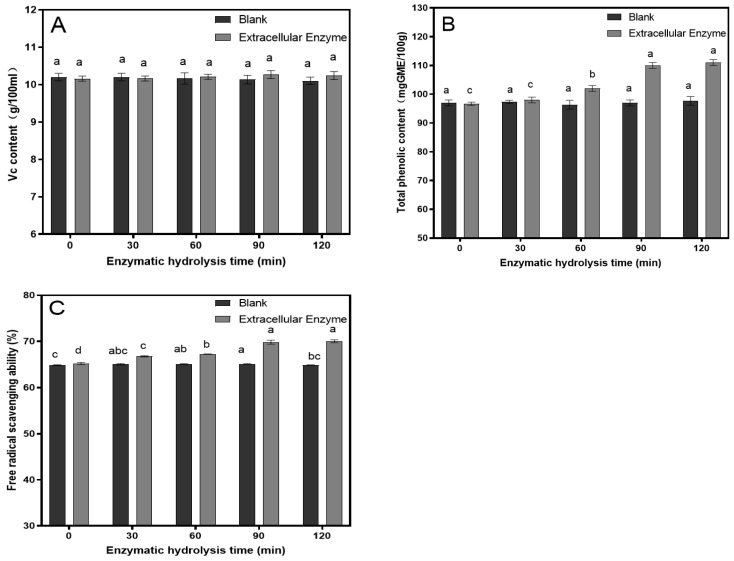
Changes in ascorbic acid (VC) (**A**), total phenol content (TPC) (**B**) content and DPPH radical scavenging ability (**C**). Different lowercase letters above the bars (a, b, c, d) indicate statistically significant differences between samples. The significance level was set at 0.05.

Ascorbic acid and phenolic compounds in fruits and vegetables exhibit multiple biological functions, including antioxidant, anti-aging, cardiovascular protective, and immune-enhancing effects. These compounds serve as natural antioxidants, contributing to the preservation of the color and flavor of fruits and vegetables, and are regarded as critical parameters in assessing food’s nutritional quality. Additionally, the concentration of phenolic compounds is often used to evaluate the physical condition and potential degradation of fruit products, such as browning, turbidity, and precipitation [38]. The study analyzed the temporal changes in ascorbic acid and total phenolic content (TPC) in pomegranate juice under the conditions of tannase addition (33.9 U/100 mL) and an enzymatic hydrolysis temperature of 30 °C. The results, as illustrated in the accompanying Figure 6, indicate that the ascorbic acid content in pomegranate juice remains relatively stable over time. In contrast, the TPC increases significantly within the first 90 min of enzymatic hydrolysis, peaking at a value of 110. After 90 min, the rate of increase in TPC diminishes, suggesting a plateau in the phenolic content. In Rababah, according to the literature reports, this content has reached a moderate level [39].

In the field of food nutrition research, the evaluation of antioxidant activity is an important indicator. It provides useful information about the health-promoting potential of a food product without requiring an analysis of each individual antioxidant component. In this study, the antioxidant activity of pomegranate juice at different enzymatic hydrolysis times was assessed by measuring its DPPH radical scavenging capacity, as shown in Figure 6. The results indicate that under the conditions of 33.9 U/100 mL tannase addition and an enzymatic hydrolysis temperature of 30 °C, the DPPH radical scavenging capacity of the pomegranate juice increased significantly before 90 min of hydrolysis, reaching 69.8% at 90 min. After 90 min, the DPPH radical scavenging capacity did not show any further significant increase. The free radical scavenging capacity in this study showed a significant improvement of nearly 20% compared to the range of 30–50% reported by RT et al. [40]. The improvement in the juice’s antioxidant capacity may be related to the increase in phenolic compounds.

### 3.4. Results of Sensory Evaluation

Color, taste, flavor, and acceptability are critical indicators for the consumer evaluation of juice products. Pomegranate juice without the addition of tannase exhibits a relatively deep color and pronounced bitterness, which may reduce its acceptability among consumers. In contrast, the pomegranate juice beverage prepared under the experimental conditions displays a bright, uniform color, with a mild taste and balanced acidity. The characteristic flavor is distinctly noticeable, complemented by a pleasant level of bitterness. As shown in Table 4, in terms of color, as the enzymatic hydrolysis time increased, the pomegranate juice gradually turned a light brown, resulting in slightly lower scores. Regarding taste, the juice developed a pleasant balance of sweetness and acidity over time, achieving the highest scores. Additionally, with longer enzymatic hydrolysis, the flavor and acceptability of the juice showed significant improvement, with both attributes receiving higher scores at 90 min, making the juice more popular among consumers.

## 4. Conclusions

This study investigated the impacts of tannase treatment conditions on the physicochemical properties and quality attributes of pomegranate juice during production. Our systematic analysis of factors like temperature, time, and enzyme dosage showed that 30 °C, 90 min, and 33.9 U/100 mL tannase levels gave an optimal juice yield, as well as optimal punicalagin, ellagic acid, and gallic acid contents. Moreover, the content of ascorbic acid, total phenols, and DPPH radical scavenging activity in pomegranate juice significantly increased under these conditions, with no further significant increase observed after 90 min of enzymatic hydrolysis. Sensory evaluation revealed high scores in terms of color, aroma, taste, and overall acceptability, with taste receiving the highest score. Considering the effectiveness of tannase in reducing astringency, along with the results for ascorbic acid, total phenols, DPPH radical scavenging activity, and sensory evaluation, the optimal processing conditions were determined to be a tannase dosage of 33.9 U/100 mL, an enzymatic hydrolysis time of 90 min, and a hydrolysis temperature of 30 °C. Overall, connecting the optimized parameters to the observed effects enables the tailored modulation of nutrition, functionality, and bioactive components in pomegranate juice. The findings of this study contribute actionable guidelines for manufacturers to enhance yields, determine enzyme impacts, and introduce novel quality control technology. The further exploration of health attributes using these optimized conditions could expand pomegranate juice functionality.

## Figures and Tables

**Table 1:** Orthogonal experimental conditions.

Level	Factor		
	A Time/(min)	B Temperature/°C	C Enzyme Dosage/(U: 100 mL)
1	75	29	27.7
2	90	30	30.8
3	105	31	33.9

**Table 2:** Sensory evaluation criteria.

Project	Scoring Criteria	Full Marks
Color	Aurantiacus, Uniformly distributed	10
Taste	Moderately sweet and sour, smooth texture	10
Flavor	Mild fruit and vegetable aroma	10
Acceptability	Degree of liking	10

**Table 3:** Orthogonal experimental results.

Experiment Number	Factor				Punicalagin Concentration (g/L)	Ellagic Acid Concentration (g/L)	Gallic Acid Concentration (g/L)
	A Time/min	B Temperature/℃	C Enzyme Dosage/ (mL: 100 mL)	D Blank			
1	1	1	1	1	3.927	0.130	2.357
2	1	2	2	2	3.808	0.132	2.362
3	1	3	3	3	3.805	0.137	2.576
4	2	1	2	3	3.704	0.142	2.382
5	2	2	3	1	3.346	0.143	2.346
6	2	3	1	2	3.854	0.131	1.896
7	3	1	3	2	3.660	0.139	2.478
8	3	2	1	3	3.566	0.137	2.484
9	3	3	2	1	3.610	0.133	2.446
K1j	3.847	3.764	3.782				
K2j	3.635	3.573	3.707				
K3j	3.612	3.756	3.604				
R	0.235	0.191	0.178				
k1j	0.132	0.137	0.132				
k2j	0.139	0.138	0.135				
k3j	0.136	0.134	0.140				
R	0.007	0.004	0.008				
k’1j	2.432	2.406	2.246				
k’2j	2.208	2.397	2.396				
k’3j	2.469	2.306	2.467				
R	0.261	0.100	0.221				

**Table 4:** Sensory evaluation results of different enzymatic hydrolysis times.

	Time/min	0	30	60	90	120
Project						
Color		Bright (7.24 ± 0.6)	Bright (7.20 ± 0.4)	Bright and uniform (7.12 ± 0.3)	Bright and uniform (7.02 ± 0.3)	Bright but slightly deep (6.80 ± 0.4)
Taste		Distinct bitterness (5.40 ± 0.3)	Distinct bitterness (6.13 ± 0.4)	Medium bitterness (7.48 ± 0.5)	Mild astringency (8.53 ± 0.2)	Mild astringency (8.60 ± 0.5)
Flavor		Marked characteristic flavor (7.79 ± 0.4)	Marked characteristic flavor (7.20 ± 0.3)	Marked characteristic flavor (6.87 ± 0.3)	Slightly reduced characteristic flavor 6.80 ± 0.4)	Diminished distinctive flavor 6.58 ± 0.2)
Acceptability		Unacceptable (4.48 ± 0.2)	Slightly acceptable (5.24 ± 0.4)	Moderately acceptable (6.93 ± 0.6)	Acceptable (7.92 ± 0.3)	Acceptable (7.94 ± 0.2)

## Data Availability

The data presented in this study are available on request from the corresponding author. The data are not publicly available due to privacy.
